# Prevalence of Oral and Maxillofacial Trauma in Elders Admitted to a Reference Hospital in Northeastern Brazil

**DOI:** 10.1371/journal.pone.0135813

**Published:** 2015-08-19

**Authors:** Marcus Antonio Melo Carvalho Filho, Maria Vieira de Lima Saintrain, Rita Edna da Silveira Dos Anjos, Solange Sousa Pinheiro, Luciana de Carvalho Pádua Cardoso, Jean André Hervé Moizan, Andréa Silvia Walter de Aguiar

**Affiliations:** 1 University of Fortaleza (UNIFOR), Collective Health Master’s Degree Program, Center of Health Sciences, Fortaleza, Brazil; 2 University of Fortaleza (UNIFOR), Center of Health Sciences, Fortaleza, Brazil; 3 University of Rouen, Dentistry Department, Rouen University Hospital, Rouen, France; 4 Federal University of Ceará, Dental Clinics Department, Fortaleza, Brazil; University of Brescia, ITALY

## Abstract

**Objective:**

To know the prevalence and etiology of oral and maxillofacial trauma in elders.

**Methods:**

Analytical quantitative cross-sectional study conducted at a public trauma hospital located in Fortaleza-Ceará, Brazil. The study population comprised patients with trauma who were hospitalized from April to August 2014. Of these patients, patients with oral and maxillofacial trauma were chosen to be included in the research. A questionnaire was administered in order to obtain information on socio-demographics, systemic comorbidities, use of medication, deleterious habits (smoking and alcohol consumption), etiology of oral and maxillofacial trauma and type of pre-hospital care.

**Results:**

Of the 280 elderly hospitalized with trauma, 47 had oral and maxillofacial trauma, with a prevalence of 16.8%. In this group, the age ranged from 60 to 88 years, with a mean age of 72.4 years (SD± 8.38). The elderly were mostly women (55.3%), self-declared *pardos* (53.2%), who presented with cardiovascular disorders (48.9%), and who received formal pre-hospital care (70.2%). Elderly who were in the 60–69 years age group, spent 6–9 years at school and drank alcohol were 2.64, 3.75, and 1.97, respectively, more likely to suffer oral and maxillofacial trauma. The main causes of trauma were physical aggression, traffic accidents, falls and domestic accidents. All of the physical aggressions resulted in oral and maxillofacial traumas, and the elderly who suffered traffic accidents were four times more likely to have oral and maxillofacial trauma.

**Conclusion:**

The prevalence of 16.8% and the lack of research on oral and maxillofacial traumas in the elderly is worrisome and should be included in the oral health indicators for the elderly population to support the importance of oral health.

## Introduction

The proportion of elderly worldwide is changing the distribution and shape population pyramid and bringing new social and economic concerns. This population is projected to reach two billion by 2050, with 80% in developing countries [1].

As one of these countries, Brazil’s demographic pattern has been undergoing deep changes due to mortality and fertility declines, leading to an increase in the life expectancy of Brazilians to 74.6 years in 2012 [2].

Unlike developed countries, Brazil aged very fast and did not subsidize social and economic changes, which challenged the country to face a new population with an epidemiological profile characterized by chronic, degenerative and disabling diseases [3, 4].

Among disabling diseases, traumas have been a growing concern for the elderly, especially because of specific characteristics, such as the decline in physiological reserves and the ability to maintain homeostasis. Older adults are more vulnerable because aging at the cellular level reflects anatomic and functional changes, which are also influenced by associated diseases and the chronic use of medications [5].

Traumas are included in the external causes category, and according to the World Health Organization in 2008, the mortality rate due to road traffic accidents, falls, unintentional injuries, self-inflicted injuries and violence accounted for 4,182 million deaths worldwide [6].

In 2010, Brazil’s National Unified Health System (SUS) registered 929,893 hospitalizations due to external causes, which accounted for 10.4% of total hospitalizations. Additionally, in 2011, older adults represented the third highest proportion for this event, accounting for 16.7% of the total [7].

Among the different types of traumas, there is complex oral and maxillofacial trauma, which includes injuries to soft tissues, teeth, bones, and vital structures, such as the brain, and sensory structures, such as the nose and eyes that require multidisciplinary treatment including oral and maxillofacial surgery, ophthalmology, plastic surgery, neurosurgery, and others [8].

One study suggests that the elderly have more porous bones, which leaves them susceptible to fractures, especially in the case of edentulous mandibles, which are reduced because of resorption of the mandibular alveolar bone. Additionally, according to this same study, facial trauma has not been properly researched in developing countries, making it difficult to obtain studies relating geriatrics to oral and maxillofacial surgery [9].

In fact, oral and maxillofacial trauma among the elderly constitutes a public health concern involving sequelae, suffering, and aesthetics, which affect well-being and self-esteem; thus, there is an interest in contributing to the existing body of knowledge, with an investigative approach to document the prevalence and etiology of oral and maxillofacial traumas and relating them to other types of accident-related traumas in hospitalized elders in an oral and maxillofacial traumatology and surgery service.

Based on these features, it will be possible to determine the epidemiological profile of these patients, making this research both academically and socially relevant. Healthcare professionals, caregivers and families are increasingly expected to become aware of the importance of health promotion and to be able to avoid patients’ isolation due to disease processes resulting from such accidents. The findings of this study are intended to stimulate further research on this issue and the development of public policies aimed at caring for the elderly.

## Materials and Methods

This is an analytical quantitative cross-sectional study conducted in a public hospital in the municipality of Fortaleza, located in the State of Ceará, Brazil. The hospital, *Instituto Dr*. *José Frota (IJF)*, was founded in 1940 and became a municipal autarky in 1970. It is considered a center of excellence and a national referral center for trauma, urgency and emergency. Its nine-story building has 425 beds, where an average 15,500 monthly admissions are attended by a multi-professional team of 2,191 clinicians and staff from different specialty areas.

Its functional structure includes an imaging center, emergency rooms, a minor operation room, a resuscitation room, rooms for critical and stabilized patients (Risk I and Risk II), a burn treatment center, wards, specialized emergency rooms (oral and maxillofacial surgery, respiratory and digestive endoscopy, ophthalmology, and otorhinolaryngology), an operating room, four intensive care units, echocardiogram, hematology unit and blood and hemoderivate bank, toxicology care center, social work and hospital psychology services aimed at the prevention and care of child and adolescent maltreatment, and an Intra-hospital Commission on Organs and Tissues Donation for Transplant.

The study population comprised elderly patients who were hospitalized in the five trauma units of the hospital during the period April to August 2014. The population also included a census of patients hospitalized with oral and maxillofacial trauma. According to Brazil’s National Health Policy for the Elderly, an older person is anyone 60 years of age or older [10].

Data collection involved two stages. First, secondary information was obtained from the hospital information system using daily data, organized by unit and bed of the five hospitalization units. This census provided the number of all patients admitted to the hospital care system. After this information (patient, age, type of trauma) was transcribed, a questionnaire was used to obtain data on sex, race, marital status, education, origin (capital or other municipalities in the State of Ceará), income, presence of systemic comorbidities, use of medication, smoking and alcohol consumption, and specific information, such as oral and maxillofacial trauma etiology, and the type of prehospital care (formal or informal).

The study included patients aged 60 years and older, who were hospitalized with trauma, who agreed to participate in the research, and who presented with the physical (capacity to write and speak) and mental capacity (temporal and spatial consciousness) to answer the questionnaire. If patients did not present with such abilities, their caregivers, nurses or families could answer the questionnaires. However, all participants were able to answer the questionnaire without the need for someone to answer it on their behalf. A consent form was signed either by the participant or by their next of kin (in the case of illiterate individuals), who were aware of the objectives and procedures. Anonymity of participants and confidentiality of information were guaranteed.

This research is in accordance with all ethical standards, and the project and consent procedures have been approved by the Research Ethics Committee of the University of Fortaleza (UNIFOR) under Protocol No. 564.088/2014.

Data were analyzed using the Statistical Package for the Social Sciences—SPSS version 21 (SPSS Inc., Chicago, IL, USA). Statistical measures, such as Pearson’s Chi-squared test, Fisher’s Exact test and Odds Ratio (OR), allowed researchers to achieve research goals with the significance levels set at 5%.

## Results

In order to estimate prevalence and obtain information on oral and maxillofacial trauma in the elderly, interviews were carried out with 280 elderly patients with different types of trauma. Within this group, 47 patients had oral and maxillofacial trauma; a prevalence of 16.8%.

Concerning the total initial population (280 elderly patients), the mean age was 76 years (SD ± 9.0), ranging from 60 to 99 years.

The main characteristics of the study population were 70–79 years of age (35.4%), predominantly female (61.8%), self-declared white (49.6%), received up to two minimum wages (87.9%), presented with cardiovascular disorders (52.9%), had formal pre-hospital care (64.3%), and suffered from domestic accidents (63.6%).

The age of the elderly with oral and maxillofacial trauma (n = 47) ranged from 60 to 88 years, with a mean age of 72.4 years (SD± 8.38). They were mostly female (55.3%), were self-declared *pardos* (mixed race Brazilians whose color is intermediate between “black” and “white”) (53.2%), had cardiovascular disorders (48.9%), had received formal pre- hospital care (70.2%), and had suffered traffic accidents (46.8%).

Table 1 shows the analysis of the association between oral and maxillofacial trauma and other types of trauma with socio-demographic variables. Odds ratios for the 60–69 years age group, 6–9 years of education, and alcohol consumption were 2.64, 3.75, and 1.97, respectively, for oral and maxillofacial trauma. Smoking was the only significant variable (p = 0.024), with 2.25 higher odds of presenting with this type of trauma.

**Table 1:** Socio-demographic variables among elders with oral and maxillofacial trauma (n = 47) and other traumas (n = 233). Fortaleza-Ceará, 2014.

Variables	Oral and Maxillofacial Trauma		Other Traumas		OR	CI (95%)	p value <0.05
	N	%	N	%			
**Age group**							
60–69 years	18	22.5	62	77.5	2.64	1.14–6.11	0.058
70–79 years	19	19.2	80	80.8	2.16	0.95–4.92	
80 and older	10	9.9	91	90.1	1.00	-	
**Sex**							
Male	21	19.6	86	80.4	1.38	0.73–2.6	0.317
Female	26	15.0	147	85.0	1.00	-	
**Origin**							
Municipality of Ceará	29	19.2	122	80.8	1.47	0.7–2.79	0.241
Fortaleza	18	14.0	111	86.0	1.00	-	
**Marital status**							
No companion	28	17.4	133	82.6	1.11	0.59–2.10	0.752
Companion	19	16.0	100	84.0	1.00	-	
**Race**							
Black	03	16.7	15	83.3	1.26	0.33–4.78	0.337*
*Parda*	25	20.3	98	79.7	1.61	0.84–3.10	
White	19	13.7	120	86.3	1.00	-	
**Retired**							
No	08	23.5	26	76.5	1.63	0.69–3.87	0.262
Yes	39	15.9	207	84.1	1.00	-	
**Income**							
None	02	20.0	08	80.0	1.26	0.26–6.17	0.822*
2–5 minimum wages	06	17.6	28	82.4	1.08	0.42–2.79	
1 minimum wage	39	16.5	197	83.5	1.00	-	
**Education (years)**							
None	16	16.0	84	84.0	1.19	0.36–3.89	0.195*
Up to 5 years	21	15.6	114	84.4	1.15	0.36–3.65	
6–9 years	06	37.5	10	62.5	3.75	0.87–16.19	
10 years or more	04	13.8	25	86.2	1.00	-	
**Use of medication**							
No	15	20.3	59	79.7	1.46	0.73–2.9	0.280
Yes	30	14.9	172	85.1	1.00	-	
**Deleterious habits**							
Alcohol consumption							
Yes	09	26.5	25	73.5	1.97	0.85–4.55	0.107
No	38	15.4	208	84.6	1.00	-	
Smoking							
Yes	14	27.5	37	72.5	2.25	1.1–4.6	0.024
No	33	14.4	196	85.6	1.00	-	

Source: research data.

*Fisher’s Exact Test

The health condition of these older adults with oral and maxillofacial trauma was not different from those with other types of trauma.

Physical aggression, traffic accidents, falls and domestic accidents were the causes of trauma.

Table 2 shows that all of the elders who suffered physical aggression (n = 5) presented with oral and maxillofacial trauma and that the chance of having trauma from a traffic accident is four times higher than from other causes.

**Table 2:** Etiologies of oral and maxillofacial trauma (n = 47) and other types of trauma (n = 233). Fortaleza-Ceará, 2014.

Variable	Oral and Maxillofacial Trauma		Other Traumas		OR	CI (95%)	p value
	N	%	N	%			
**Physical aggression**							
Yes	05	100.0	-	0.0	-	-	<0.001*
No	42	15.3	233	84.7	-	-	
**Traffic accident**							
Yes	22	34.4	42	65.6	4.00	2.06–7.77	<0.001
No	25	11.6	191	88.4	1.00	-	
**Falls**							
Yes	05	15.2	28	84.8	1.00	-	0.789
No	42	17.0	205	83.0	1.15	0.42–3.14	
**Domestic accident**							
Yes	15	8.4	163	91.6	1.00	-	<0.001
No	32	31.4	70	68.6	4.97	2.53–9.75	

Source: research data.

*Fisher’s Exact Test

## Discussion

This study seeks to fill a gap in the oral health care of older people because there is little information available in the literature concerning oral and maxillofacial trauma in this population.

With regard to the different types of trauma from external causes, the National Information System shows that these causes accounted for 16.7% of hospitalizations and were the sixth major cause of death in 2011 [11]. Given this information, there was a need for further exploration of this issue, which is already reflected in public health statistics and thus justifies the development of this research.

The research conducted at *IJF* for four months assessed 280 patients with trauma from external causes and verified a prevalence of 16.8% for oral and maxillofacial trauma. This trauma prevalence is similar to the 15.2% found for the facial area from studies conducted by Yildiz *et al*. [12].

For elderly participants in the initial group, both mean age and age group are similar to those of Gowing and Jain [13]. The highest prevalence of trauma is found among women (61.8%) and is supported by the Brazilian studies conducted by Lima and Campos [3] and Rodrigues and Ciosak [5]. However, in different parts of the world, like the U.S. and Turkey, the highest prevalence is among men [14, 15]. In Mexico, as in other countries, men are more likely to die from trauma than women, 23% versus 7%, respectively [16].

In the present study, the high prevalence of trauma among women may be explained by three key points: a) In Brazil, there is a feminization of aging. In 2013, the number of elderly women reached 14.5 million, while the number of elderly men totaled 11.5 million. Additionally, in 2012, life expectancy at birth was 78.3 years for women and 71.0 for men. Therefore, the greater longevity of women can increase the chances of suffering trauma events [17]; b) patients who suffered trauma from domestic accidents, especially falls, were mostly women. Authors have pointed out the high prevalence of female gender in cases of trauma from falls, as justified by the fact that women do most of the housework and are more exposed to this type of accident than men [3,5,6,15,18–20]. c) Another factor may be the medical attendance scheme in hospital trauma units. In the female unit, most of the women were waiting for a bed in the *Hospital da Mulher* (Woman’s Hospital), which supports *IJF*, in order to have their surgeries. The referral of patients generates a rotation that allows for the admission of new patients, which may justify the high number of women in the present study.

The prevalence of oral and maxillofacial trauma according to age group was similar in the 60–69 years and 70–79 years age groups. These data are corroborated by Chrcanovic, Souza and Freire-Maia [19], who did not find any differences between the two age groups. However, Iwaki-Filho *et al*. [9] and Thóren *et al*. [20] found a higher number of occurrences in the 60–69 years age group, while Paes *et al*. [21] revealed a higher prevalence in the 61–70 years age group.

Although the p value was above 0.05, notwithstanding this marginal value, the age group inference in the elderly with different types of trauma (n = 233) and in those with oral and maxillofacial trauma (n = 47) presented a statistical relationship, as the minimum confidence interval was above 1.00. Additionally, older adults ages 60–69 years were 2.64 times more likely to suffer oral and maxillofacial trauma than patients ages 80 years or older. According to Iwaki-Filho *et al*. [9], the young-elderly perform more activities and are more active, compared to the old-elderly. Moreover, in order to increase family income, they perform heavy and hazardous work that exposes them to accidents that can result in traumatic injuries.

Concerning gender, in both trauma situations, the prevalence of oral and maxillofacial trauma in the female gender was 55.3% and 63.1% for other types of traumas. This fact, justified by the feminization of aging and the attendance scheme of the hospital, is supported by the World Health Organization [16], which evidences the interaction between biological and social determinants of health and reveals that the inequity between the sexes increases exposure and vulnerability to risks, limits access to care and information, and influences health status outcomes. However, part of the literature on trauma shows a prevalence among men ranging from 51.4% to 77.5% [9, 20, 22–26].

Studies conducted with oral and maxillofacial trauma patients of all ages also reveal a predominant prevalence of male gender; however, this prevalence is lower among the elderly. The authors explain that women do most of the housework, while men perform dangerous activities, like driving on motorways and drinking more alcohol [22, 27–29].

Most of the elderly with oral and maxillofacial trauma (61.7%) came from other municipalities in the State, which caused hospital overcrowding. Despite this occurrence, two hospitals located in the municipalities of Sobral and Juazeiro do Norte strengthened the regionalization of health care and improved access for oral and maxillofacial trauma patients, who travel nearly 500 km to have surgeries in *IJF* [29]. However, there is high demand at the hospital, revealing the need to strengthen the regionalization of health care and to stimulate the decentralization of hospital access. Thus, the *IJF* could plan and organize its services more optimally and easily deliver service excellence.

In Brazil, a low-income population refers to people who receive less than two minimum wages (1MW ± 260 US$), which included most of the elderly participating in the study. However, elderly aged 65 years and older who are unable to provide for their own living and whose families are unable to provide for their living, are guaranteed one minimum wage per month [30].

The elderly who have never been to school and those who have spent only five years at school accounted for 78.7% of the patients with oral and maxillofacial trauma and 85.0% of patients with other types of trauma, respectively. Income and education associated with socioeconomic inequalities may have interfered with users’ search for public care in the trauma center of *IJF*.

This finding is corroborated by research conducted in England, Wales and Northern Ireland that found a significant association (p<0.001) between socioeconomic inequalities and major impacts on the general and oral health of low-income adult participants when compared to high-income participants [31].

Although there was no significant difference between living with a companion and suffering oral and maxillofacial trauma or other types of trauma, the prevalence of trauma among the elderly living with companions was lower than that of the elderly who in both cases had no companions, 40.4% *vs* 59.6% and 42.9% *vs* 57.1, respectively. Noteworthy is the study by Reis *et al*. [32] that revealed that family support contributes to the maintenance of physical and psychological integrity, reinforcing what is preconized by Brazil’s National Health Policy for the Elderly [10].

Regarding self-reported diseases, cardiovascular disorders were not significantly associated with trauma; however, they were widely reported (48.9%) and are in accordance with World Health Organization reports that reveal they are responsible for 10% of the “disability-adjusted life years” lost in low and middle-income countries [33].

Diabetes mellitus was reported by 14.9% of the elders, a value that is similar to the 13.7% detected in the Brazilian population [4]. Considered a current worldwide epidemic, the number of cases of diabetes in 1985 was estimated at 30 million adults, increasing to 173 million in 2002. This figure will probably reach 300 million cases by 2030, and nearly two thirds of the people with diabetes mellitus live in developing countries [34]. Health issues like vision, auditory and speech problems were widely reported in the present research (72.3%, 23.4% and 17%, respectively) and may favor the occurrence of trauma. It is important to mention that there may be additional diseases among the elderly participating in the research, as they did not often recognize their real health status.

The most common type of hearing loss among elderly people is bilateral sensorineural hearing loss, which compromises the speech intelligibility threshold. The incidence of presbycusis tends to increase, and further studies are required specifically of the elderly population, in order to facilitate intervention and promotion actions [35].

Considering vision problems, Ordinance No. 958/2008 of the Ministry of Health of Brazil redefines the National Policy for Medium Complexity Elective Surgical Procedures [36] and recognizes them as public health concerns, due to the high incidence of vision impairment in the Brazilian population (18.8%) [2].

In addition to health problems, the use of medications was also evident. Overall, 63.8% of elderly with oral and maxillofacial trauma and 73.8% of elderly with other types of trauma used some type of medication. These figures are similar to those found by Lima and Campos [3]; however, they are higher than the 45.3% found by Iwaki-Filho *et al*. [9].

The elderly’s deleterious habits like drinking alcohol (19.1%) and smoking (29.8%) can also influence the occurrence of trauma. From our study data, elderly smokers were 2.25 times more likely to suffer oral and maxillofacial trauma. Zaitune et al. [37] observed that older smokers are more exposed to complications and to a high prevalence of severe diseases. Additionally, they tend to have lower body weight and exercise less. Therefore, they become fragile and hence more susceptible to trauma.

Of all the oral and maxillofacial trauma cases, 46.8% were caused by traffic accidents. People involved in this type of accident had four times greater risk of oral and maxillofacial trauma when compared to the other causes.

Studies by Rahman *et al*. [38] and Paes *et al*. [21] also showed prevalence of traffic accidents, but with higher percentages, 64.2% and 52.5%, respectively. This type of accident could easily be avoided. However, in Brazil, drivers and riders do not respect the law, exceed speed limits, drink and drive, and do not wear helmets, causing very severe accidents.

In the present study, the elderly’s traumas from falls also became an important public health concern. Between 1990 and 2010, there was a significant increase in the number of cases of falls among the elderly [39]. Additionally, 40% of elderly patients who suffered oral and maxillofacial trauma from falls presented with multiple fractures and needed to spend longer time in hospital. They also presented with greater morbidity and mortality [40]. Other studies conducted with older adults who suffered oral and maxillofacial trauma pointed to falls as the main etiology [9,20,25,26].

It is important to mention that all cases of physical aggression resulted in oral and maxillofacial trauma, with 80% of the cases occurring among men. Although alcohol did not appear as a risk factor in the present study, its consumption may lead to quarrels and physical aggression, with facial aggression standing out as the main act of this behavior [27]. Moreover, men tend to drink more alcohol than women, which may justify the male gender prevalence.

The absence of a significant statistical relationship between the elderly who used medications and those who suffered oral and maxillofacial traumas and other types of traumas may be evidenced by the fact that traumas are not necessarily associated with manifested pathologies. For instance, patients with undiagnosed and untreated osteoporosis have fragile bones and are, hence, more susceptible to falls and fractures.

Although the use of medication has not interfered with the findings, considerations on dentistry and elderly patients are very important. Elderly people present a greater accumulation of multiple diseases that requires the use of different medications and a complex approach. To do so, dentist-surgeons should be highly qualified to deliver special and distinguished care, sophisticated anamnesis, high-accuracy clinical exams and detailed analysis of complimentary exams. They should also have a good interpersonal relationship with other professionals in a multidisciplinary and interdisciplinary way in order to ensure safe care. The success of the dental treatment of elders may be compromised if these factors are neglected [41].

Although the hospital, professionals and patients were very open to the research team, there were some limitations to the conduct of the study, such as the registration of traumas with unspecified diagnosis and the suppression of registrations about real health status, because the elderly and caregivers were not aware of it. Additionally, some information could not be explored because of the elderly patients’ fragility.

Because this study was limited to a trauma hospital, further studies are needed to improve research on this issue. However, as it has been conducted in a large hospital that delivers service excellence to Northern and Northeastern Brazil, its results should probably be similar to results of different places and could contribute to the planning of actions targeted at the prevention and rehabilitation of trauma among people of all ages.

## Conclusion

The present study revealed a 16.8% prevalence of oral and maxillofacial trauma among the elderly, especially among the young elderly, a fact that should be included in oral health indicators for the elderly population.

Traffic accidents were perceived as the major etiology for the oral and maxillofacial trauma among the elderly; the elderly who suffered traffic accidents were four times more likely to have oral and maxillofacial trauma.

Based on these findings, the prevalence observed among the elderly and the lack of research in this area deserve special attention from health care systems, not only because of physical and psychological problems that hinder rehabilitation but also to reinforce the means of promotion and prevention and health education in an interdisciplinary way, so that future occurrences can be avoided.
